# Author Correction: Muscimol inhibits plasma membrane rupture and ninjurin-1(NINJ1) oligomerization during pyroptosis

**DOI:** 10.1038/s42003-024-05975-3

**Published:** 2024-03-11

**Authors:** Andreas B. den Hartigh, Wendy P. Loomis, Marisa J. Anderson, Bente Frølund, Susan L. Fink

**Affiliations:** 1 Department of Laboratory Medicine and Pathology, University of Washington, Seattle, WA USA; 2 Department of Drug Design and Pharmacology, Faculty of Health and Medical Sciences, University of Copenhagen, Copenhagen, Denmark

**Keywords:** Cell death, Inflammation

Correction to: *Communications Biology* 10.1038/s42003-023-05354-4, published online 05 October 2023

The title of this article originally read:

“Muscimol inhibits plasma membrane rupture and ninjurin-1 oligomerization during pyroptosis”

It has now been corrected to:

“Muscimol inhibits plasma membrane rupture and ninjurin-1(NINJ1) oligomerization during pyroptosis”

