# Supplementary material for: Muscimol inhibits plasma membrane rupture and ninjurin-1(NINJ1) oligomerization during pyroptosis
Source: Commun Biol. 2023 Oct 5;6:1010. doi: 10.1038/s42003-023-05354-4 (PMC10556065; doi:10.1038/s42003-023-05354-4)
Supplement: Supplementary file 2 — Supplemental Material [file 42003_2023_5354_MOESM2_ESM.pdf]

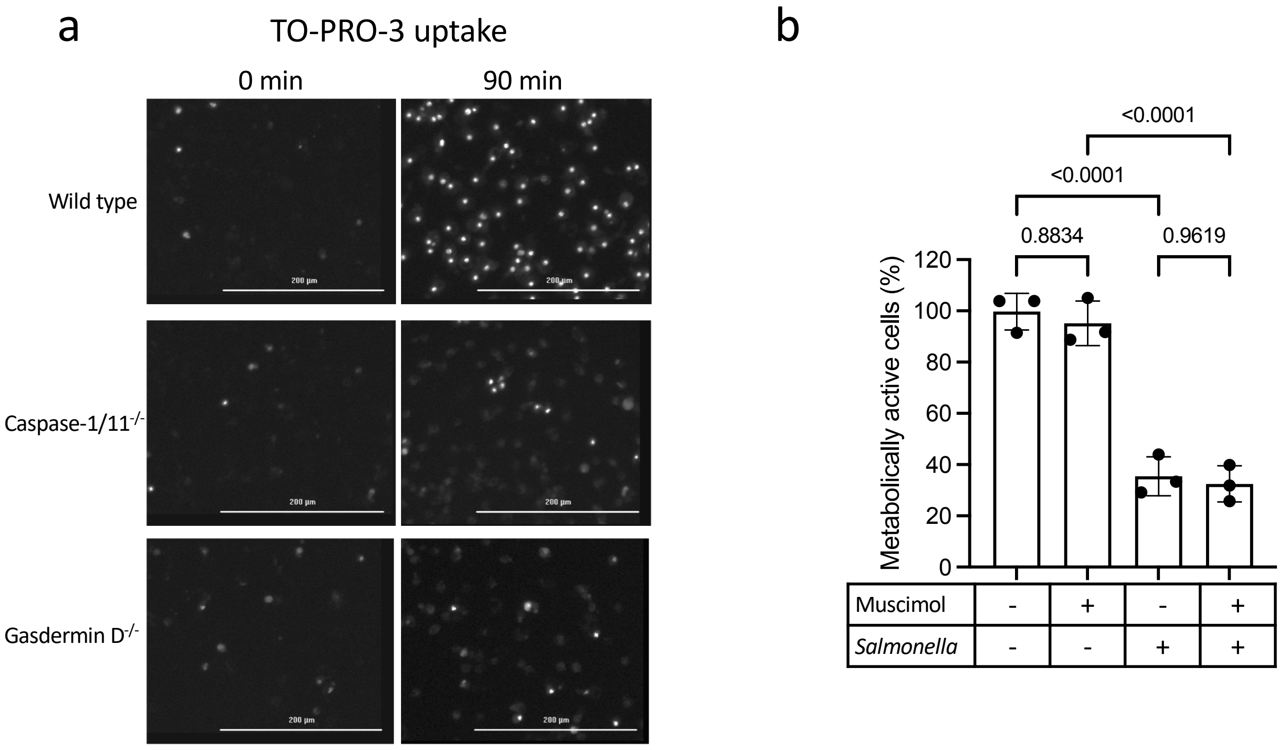

Supplemental Figure 1: Muscimol does not affect gasdermin D pore formation, nor does it alter cellular viability. (a) TO-PRO-3 uptake was assessed in wild type, caspase-1/11$^{-/-}$, or gasdermin D$^{-/-}$ BMMs infected with *Salmonella* in the presence of muscimol. Images representative of 5 taken per condition in 3 independent experiments are shown. Scale bars represent 200 μm. **(b)** Percentage of metabolically active cells (assessed by measuring ATP content) in uninfected or *Salmonella* infected cells in the presence or absence of 1 mM muscimol was assessed. Representative data (mean ± SD, n = 3) from three independent experiments are shown.

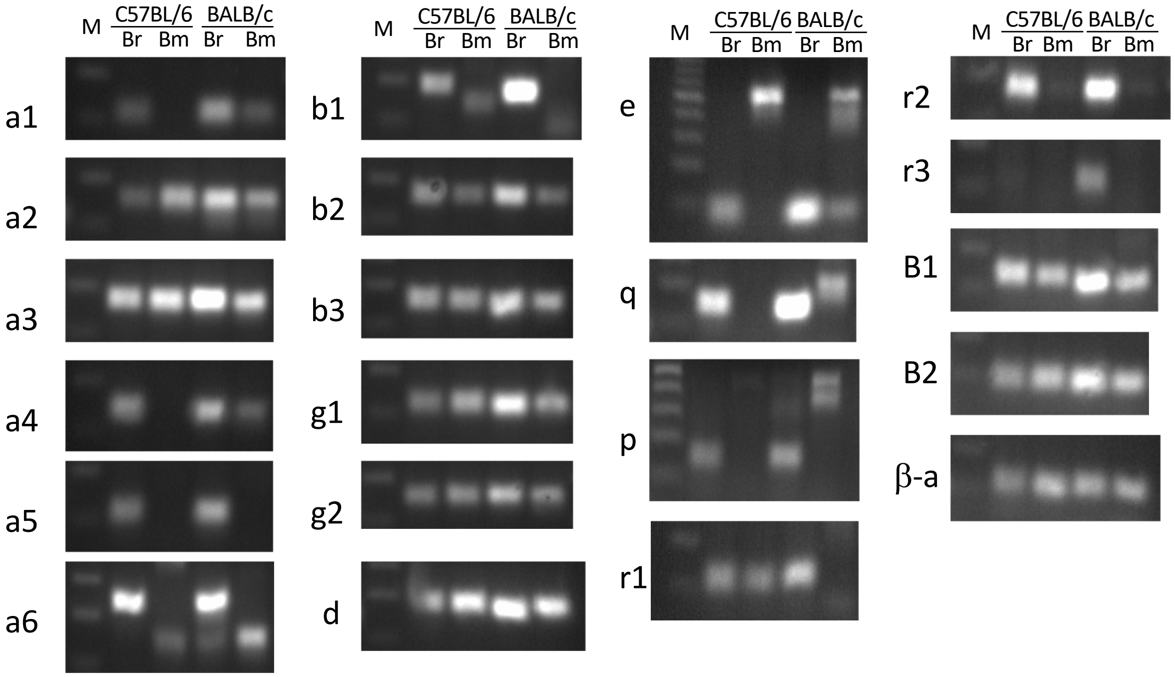

Supplemental Figure 2: Expression of GABA receptor subtypes. GABA receptor subunit expression in the brain (Br) and bone marrow macrophages (Bm) of C57BL/6J and BALB/cJ mice. RNA was transcribed into cDNA and amplified with primers listed in experimental details. Samples were separated on a 3% agarose gel and visualized with GelRed DNA dye. β-a = beta-actin

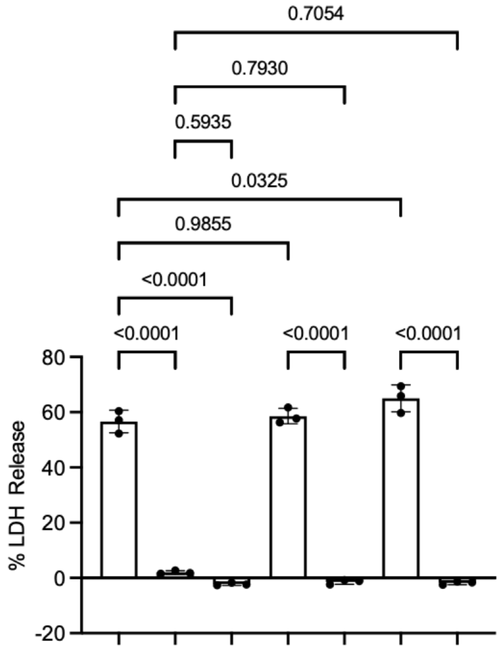

Salmonella	+	+	+	+	+	+	+
Muscimol	-	+	+	-	+	-	+
GABA	-	-	+	-	-	-	-
Gabazine	-	-	-	+	+	-	-
Picrotoxin	-	-	-	-	-	+	+

Supplemental Figure 3: Effect of GABA (ant)agonists on inhibition of cellular lysis by muscimol. BMM were infected with *Salmonella* in the presence of GABA and muscimol, Gabazine and muscimol, or Picrotoxin and muscimol as indicated. LDH released during cell lysis was measured. Representative data (mean ± SD, n = 3) from 2 independent experiments are shown. Statistics: one-way ANOVA + Tukey’s multiple comparisons.

a

Compound	FOM Number	Effect on $GABA_A$
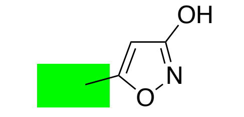	5-5	No activity
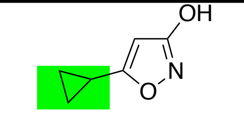	5-6	No activity
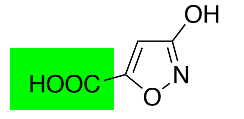	5-7	No activity
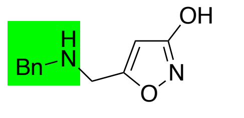	5-8	No activity
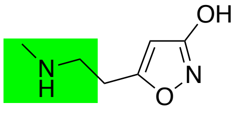	5-9	No activity

b

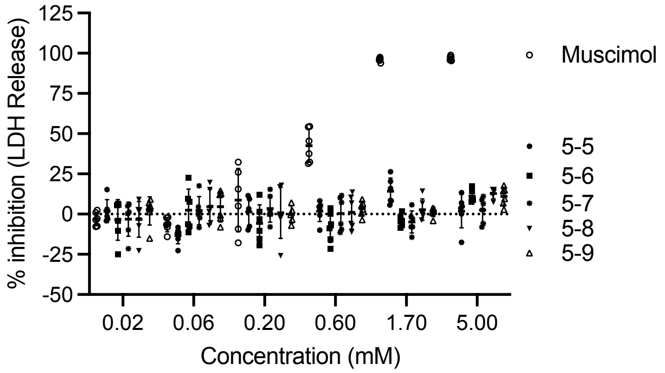

Supplemental Figure 4: Modification of muscimol at position 5 abolishes inhibitory capacity of muscimol. (a) Structures of muscimol analogs modified at position 5 of the ring structure that are inactive at the GABA receptor. **(b)** BMM were infected with *Salmonella* in the presence of indicated concentrations of muscimol or muscimol analogs. LDH released during cell lysis was measured and used to calculate inhibitory percentage. Combined data of 2 independent experiments (mean ± SD, n = 3 per experiment) are shown

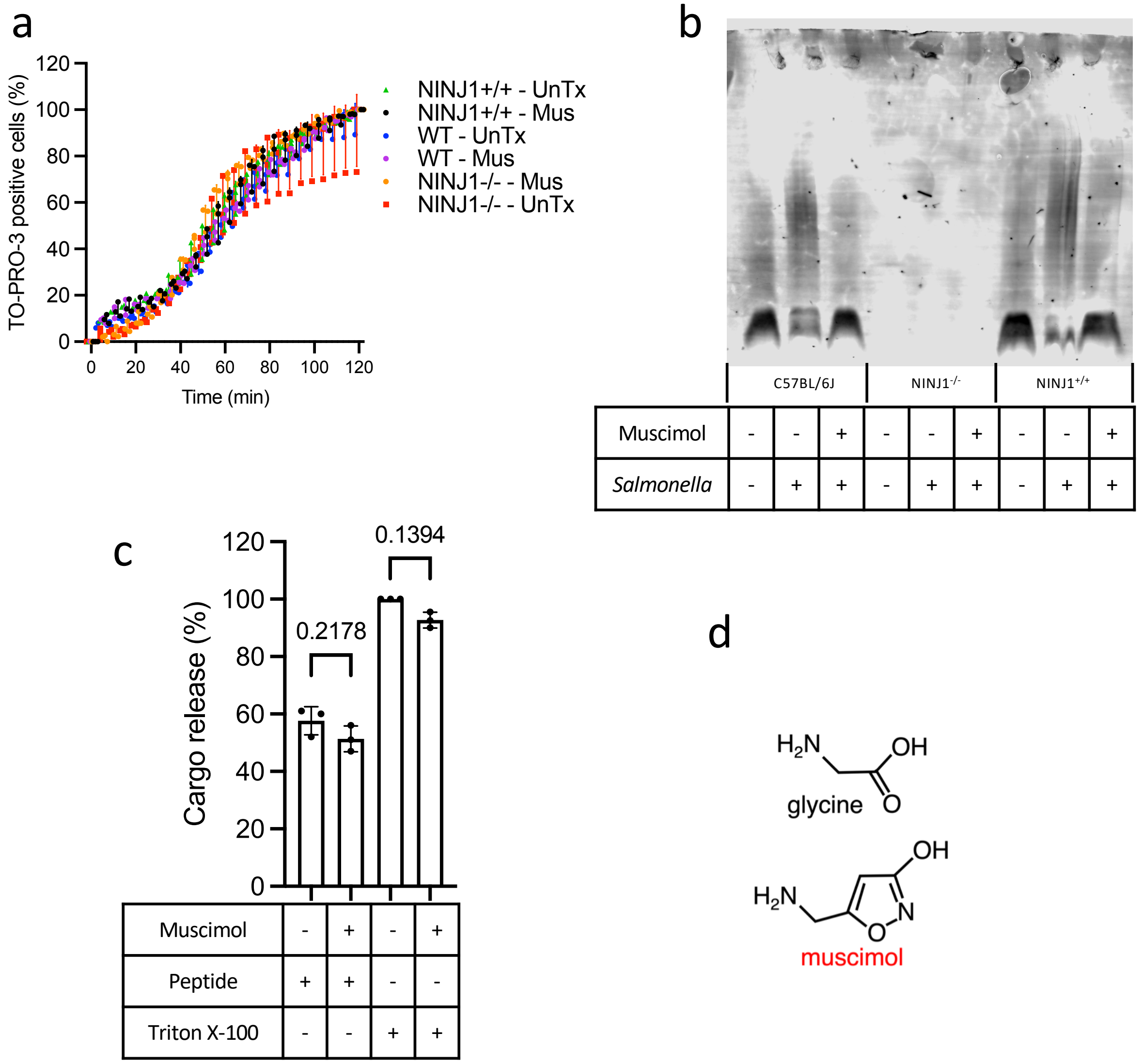

Supplemental Figure 5: (a) TO-PRO-3 uptake was assessed in C57BL/6J WT, ninjurin-1$^{-/-}$, or ninjurin-1$^{+/+}$ (C57BL/6N) BMMs infected with *Salmonella* in the presence of muscimol, as indicated. The percentage of cells with TO-PRO-3+ nuclei was quantified. **(b)** Full source Western blot corresponding to Fig 8b. BN-PAGE Western blot for ninjurin-1 oligomerization in uninfected, *Salmonella*-infected, and *Salmonella*-infected muscimol treated cells. **(c)** Release of 5(6)-Carboxy-fluorescein from liposomes exposed to ninjurin-1 peptide or Triton X-100 total lysis control in the presence of 1 mM muscimol, as indicated. **(d)** Structures of muscimol and glycine. Representative data (mean ± SD, n = 3 (a,c)) from two or three independent experiments are shown. Statistics: one-way ANOVA + Tukey's multiple comparisons.
