# Supplementary material for: Muscimol inhibits plasma membrane rupture and ninjurin-1(NINJ1) oligomerization during pyroptosis
Source: Commun Biol. 2023 Oct 5;6:1010. doi: 10.1038/s42003-023-05354-4 (PMC10556065; doi:10.1038/s42003-023-05354-4)
Supplement: Supplementary file 3 — Description of Additional Supplementary Files [file 42003_2023_5354_MOESM3_ESM.pdf]

Description of Additional Supplementary Files

File name: Supplementary Data 1
Description: The source data behind all graphs in the manuscript.
