# Supplementary material for: Muscimol inhibits plasma membrane rupture and ninjurin-1(NINJ1) oligomerization during pyroptosis
Source: Commun Biol. 2023 Oct 5;6:1010. doi: 10.1038/s42003-023-05354-4 (PMC10556065; doi:10.1038/s42003-023-05354-4)
Supplement: Supplementary file 5 — Reporting Summary [file 42003_2023_5354_MOESM5_ESM.pdf]

nature portfolio

Corresponding author(s): Susan Fink

Last updated by author(s): Aug 21, 2023

Reporting Summary

Nature Portfolio wishes to improve the reproducibility of the work that we publish. This form provides structure for consistency and transparency in reporting. For further information on Nature Portfolio policies, see our Editorial Policies and the Editorial Policy Checklist.

Statistics

For all statistical analyses, confirm that the following items are present in the figure legend, table legend, main text, or Methods section.

n/a	Confirmed	
☐	☒	The exact sample size (n) for each experimental group/condition, given as a discrete number and unit of measurement
☐	☒	A statement on whether measurements were taken from distinct samples or whether the same sample was measured repeatedly
☐	☒	The statistical test(s) used AND whether they are one- or two-sided<br>*Only common tests should be described solely by name; describe more complex techniques in the Methods section.*
☐	☒	A description of all covariates tested
☒	☐	A description of any assumptions or corrections, such as tests of normality and adjustment for multiple comparisons
☐	☒	A full description of the statistical parameters including central tendency (e.g. means) or other basic estimates (e.g. regression coefficient) AND variation (e.g. standard deviation) or associated estimates of uncertainty (e.g. confidence intervals)
☒	☐	For null hypothesis testing, the test statistic (e.g. F, t, r) with confidence intervals, effect sizes, degrees of freedom and P value noted<br>*Give P values as exact values whenever suitable.*
☒	☐	For Bayesian analysis, information on the choice of priors and Markov chain Monte Carlo settings
☒	☐	For hierarchical and complex designs, identification of the appropriate level for tests and full reporting of outcomes
☒	☐	Estimates of effect sizes (e.g. Cohen's d, Pearson's r), indicating how they were calculated

Our web collection on statistics for biologists contains articles on many of the points above.

Software and code

Policy information about availability of computer code

Data collection	Kinetic data were collected using Agilent-Biotek Cytation 1 running Gen5 v3.11. LDH and IL-1b release was determined using Molecular Devices SpectraMax M3 running SoftMax Pro v6.2.1. Confocal images were taken on a Leica SP8X confocal running LasX. Circular Dichroism was performed on a Jasco 720
Data analysis	Graphpad Prism version 9.4.1, Gen5 v3.11, and Adobe Photoshop CS5

For manuscripts utilizing custom algorithms or software that are central to the research but not yet described in published literature, software must be made available to editors and reviewers. We strongly encourage code deposition in a community repository (e.g. GitHub). See the Nature Portfolio guidelines for submitting code & software for further information.

Data

Policy information about availability of data

All manuscripts must include a data availability statement. This statement should provide the following information, where applicable:

- Accession codes, unique identifiers, or web links for publicly available datasets
- A description of any restrictions on data availability
- For clinical datasets or third party data, please ensure that the statement adheres to our policy

The data that support the findings in this manuscript are available from the corresponding author upon request.

Human research participants

Policy information about studies involving human research participants and Sex and Gender in Research.

Reporting on sex and gender	n/a
Population characteristics	n/a
Recruitment	n/a
Ethics oversight	n/a

Note that full information on the approval of the study protocol must also be provided in the manuscript.

Field-specific reporting

Please select the one below that is the best fit for your research. If you are not sure, read the appropriate sections before making your selection.

☒ Life sciences ☐ Behavioural & social sciences ☐ Ecological, evolutionary & environmental sciences

For a reference copy of the document with all sections, see nature.com/documents/nr-reporting-summary-flat.pdf

Life sciences study design

All studies must disclose on these points even when the disclosure is negative.

Sample size	No sample size calculations were performed. Sample size are based on experience performing similar experiment.<br>Statistical analysis was used to determine if statistical significance was reached with the chosen sample size
Data exclusions	No data were excluded from the analysis.
Replication	Replication was successful in all experiments performed. Either 2 or 3 independent experiments were performed with triplicate samples in each experiment.
Randomization	Randomization was only applied to the animal experiment where mice were assigned to cages at time they entered our facility. No randomization was performed in the in vitro experiments. These were setup such that the least amount of sample handling was required during the experiment to minimize variation.
Blinding	Serum samples send for analysis of BUN levels were blinded to the vendor performing the assay. All other samples were not blinded. The small size of our lab precludes using blinding during in vitro experiments.

Reporting for specific materials, systems and methods

We require information from authors about some types of materials, experimental systems and methods used in many studies. Here, indicate whether each material, system or method listed is relevant to your study. If you are not sure if a list item applies to your research, read the appropriate section before selecting a response.

Materials & experimental systems

n/a	Involved in the study	
☐	☒	Antibodies
☐	☒	Eukaryotic cell lines
☒	☐	Palaeontology and archaeology
☐	☒	Animals and other organisms
☒	☐	Clinical data
☒	☐	Dual use research of concern

Methods

n/a	Involved in the study	
☒	☐	ChIP-seq
☒	☐	Flow cytometry
☒	☐	MRI-based neuroimaging

Antibodies

Antibodies used	HMGB1: Abcam cat no ab79823, RRID:AB_1603373. Ninjurin-1 antibody was a gift from Genentech
Validation	Antibodies used were reported and validated by the manufacturer.

Validation	HMGB1: https://www.abcam.com/hmgb1-antibody-epr3507-ab79823.html<br>Ninjurin-1 antibody was gifted by Genentech and no validation data provided. PMID 33472215 Fig 4c shows specificity.

Eukaryotic cell lines

Policy information about cell lines and Sex and Gender in Research

Cell line source(s)	Bone marrow macrophages were isolated from both male and female mice (pooled). L929 cells were obtained from (ATCC Cat no CCL-1, RRID:CVCL_0462)
Authentication	No authentication was performed.
Mycoplasma contamination	Cells were not tested for mycoplasma contamination
Commonly misidentified lines<br>(See ICLAC register)	n/a

Animals and other research organisms

Policy information about studies involving animals; ARRIVE guidelines recommended for reporting animal research, and Sex and Gender in Research

Laboratory animals	Mice: C57BL/6 (stock no: 000664, RRID:IMSR_JAX:000664), C57BL/6 ASC-Citrine (stock no: 030744, RRID:IMSR_JAX:030744), Gasdermin D-/- (stock no: 032410, RRID:IMSR_JAX:032410), and BALB/c (stock no: 000651, RRID:IMSR_JAX:000651) mice were purchased from Jackson Laboratories. Caspase-1/11-/- mice were a kind gift from Dr. Richard A. Flavell, Yale University and bred on-site. Ninjurin-1+/- mice were a kind gift from Genentech and bred on-site to generate Ninjurin-1-/- and Ninjurin-1+/+ litter mate controls.
Wild animals	n/a
Reporting on sex	Sex was not considered in this study
Field-collected samples	n/a
Ethics oversight	Mice were housed in specific pathogen-free conditions according to the University of Washington Institutional Animal Care and Use Committee guidelines

Note that full information on the approval of the study protocol must also be provided in the manuscript.
